# School Nurses’ Experiences Meeting Children Who Live in a Culture of Honor: A Qualitative Interview Study

**DOI:** 10.1177/10598405251327736

**Published:** 2025-03-28

**Authors:** Malin Jakobsson, Emma Elgan, Malin Qvist, Lise-Lotte Jonasson

**Affiliations:** 1 Department of Nursing, School of Health and Welfare, Jönköping University, Jönköping, Sweden; 2 CHILD Research Group, Jönköping University, Jönköping, Sweden

**Keywords:** culture of honor, child health, school nurse, qualitative content analysis

## Abstract

The school nurse's work is to support children's health and identify those at risk of being maltreated, a situation that children living in a culture of honor may face. The aim was to describe the school nurse's experience of meeting children living in a culture of honor. The study was qualitative, using qualitative content analysis with an inductive approach. The data was collected through semistructured interviews with 10 school nurses from different parts of Sweden. The results are illustrated in an overarching theme: walking a tightrope when supporting children living in a culture of honor, and three categories. The categories are: working preventively around children, building trust in children, and collaborating around the children. Based on the study, school nurses desire regular training on the culture of honor and greater collaboration with other professional groups, especially social services, to feel more confident in their work.

## Introduction

The Convention on the Rights of the Child adopted by the United Nations (UN) in 1989, serves as a global framework to protect children's rights, requiring that all individuals working with children prioritize their best interests (United Nations, 1989). When the convention was enacted as Swedish law in 2020, protecting children's rights was strengthened (SFS, 2018:1197). However, significant challenges remain in the practical application of these rights, particularly in the context of honor-related violence and the vulnerability of children within different cultural frameworks. There are several definitions of honor-related violence and oppression; this study leans on the Swedish government's definition (Prop., 2019/20:131). “*Honor-related violence and oppression mean that people, mainly girls and women but also boys and men, are limited in their lives and exposed to pressure and violence aimed at maintaining the family's control over the individual … The control of girls and women ranges from restrictions in everyday life regarding clothing, socializing, and freedom of movement to restrictions on choices of education, work, and marriage. The consequences can be severe for those who try to defy control. In their most extreme form, honor norms can lead to serious violent crime.”*

School nurses are responsible for prioritizing the child's best interests and promptly identifying and reporting any suspicions of harm to social services (National Board of Health and Welfare, 2024; Regber, 2023). This responsibility includes recognizing signs of honor-related violence, one of the most severe forms of abuse of children's rights (Gregory et al., 2020; Harding et al., 2019). It can include domestic abuse, child abuse, female genital mutilation, forced marriage, isolation from friends and family, verbal violations, control, sexual violence, and threats (Bhanbhro et al., 2016; Gregory et al., 2020; Strid et al., 2021). The male relatives of a family set rules that protect the honor of a family or a community to preserve perceived social, cultural, or religious traditions or norms. If the woman or girl breaks the rules, she is punished for disgracing the family (Gregory et al., 2020; Strid et al., 2021). Honor-related violence is a global public health problem (Bhanbhro et al., 2016). Estimating the prevalence of children living in a culture of honor is challenging, given that the phenomenon is either kept secret, not monitored, or justified by law. As an indication of the prevalence, a study from Sweden's three largest cities showed that 14% of school students live in a culture of honor. Moreover, it showed that living with a culture of honor significantly increases exposure to physical, sexual, psychological, material, and economic violence. Higher levels of both violence and isolation were seen for girls than for boys (Strid et al., 2021). These findings show that many children in school live in a culture of honor. In this context, school nurses are important in providing attention and support. The need for school nurses’ promotional work is further manifested by studies showing associations between child maltreatment and negatively affected academic performance (Fry et al., 2018; Ryan et al., 2018).

School nurses play a vital leadership role in advocating for children's health and wellbeing in school (UNESCO, 2024). In Sweden, school nurses conduct four individual health dialogs at regular intervals over the years that children are in school (National Board of Health and Welfare & Swedish National Agency for Education, 2023; SFS, 2010:800). Health dialogs are offered to every student and last up to 1 h per session. The sessions include health checkups such as measuring height and weight, assessing vision and hearing, and screening for scoliosis. However, most of the time is dedicated to discussion. The school nurse and the child reflect upon the child's health based on a health survey completed in advance by the child. This process provides opportunities to identify various health-related problems and, at the same time, detect signs of honor-related issues.

School nurses, who play a central role in identifying and reporting concerns regarding children's welfare, frequently face obstacles such as insufficient resources, inadequate training, and a complex social reality. This complicates the identification and management of signs of violence and abuse (Harding et al., 2019; Kraft & Eriksson, 2014). The cultural dimension of honor-related violence further complicates the identification of vulnerable children, as norms and values can vary significantly among different groups (Regber, 2023). Many school nurses experience uncertainty about how to act when cultural practices conflict with children's rights. This creates a risk that children in vulnerable situations may not receive the support they need (Bhatia et al., 2024; Sundler et al., 2021).

At the same time, it is crucial to acknowledge that honor-related violence is a complex issue that cannot solely be understood as an individual problem. It is part of broader societal structures that uphold patriarchal norms and power imbalances (Gregory et al., 2020). Therefore, the challenge lies in identifying and reporting violence and creating a school and healthcare environment where all children feel safe, respected, and heard, regardless of their cultural background (Alizadeh et al., 2011; UNICEF, 2024). It is essential that school nurses are integrated into broader social initiatives and that various actors coordinate to combat honor-related violence and protect children's rights effectively (National Board of Health and Welfare, 2024; UNESCO, 2024). Deepening our understanding of the school nurses’ role and their practical difficulties can help us identify areas where additional support and resources are needed. Therefore, this study aimed to describe the school nurses’ experience of meeting children living in a culture of honor.

## Method

### Design

An inductive qualitative design was adopted to address this study's aim. A qualitative content analysis developed by Graneheim and Lundman (2004) and refined by Lindgren et al. (2020), which strives to make the participants’ voices heard, was used. The study was performed from January to March 2024. The study followed the ethical regulations and guidelines outlined in Swedish law (SFS, 2003:460), and no sensitive personal data, such as birth date, religion, race, or health status, was requested. Moreover, the study did not involve an intervention that aimed to affect the participant physically or psychologically. The Declaration of Helsinki's code of ethics was carefully followed, and the requirements of information, consent, confidentiality, and usage were fulfilled (World Medical Association, 2013). The consolidated criteria for reporting qualitative research (COREQ) were followed (Tong et al., 2007).

### Participants

In this qualitative study, a varied group of participants was desired. Purposive sampling was chosen to give the study breadth, and it included school nurses who had worked with children living in a culture of honor. The inclusion criteria for the study were that the school nurses had a Master of Science in Nursing, had worked at least 1 year as a school nurse, and had experience dealing with children living in a culture of honor. Several schools were contacted, and 10 female school nurses agreed to participate in the study. The school nurses were from a wide geographical area in Sweden and worked in all types of schools, such as primary, secondary, and upper-secondary schools. Nine of the school nurses had 3 to 10 years of working experience, and one had more than 16 years. All of them had experience of meeting children living in a culture of honor (Table 1).

**Table 1.** Overview of the Participants, Year Working as a School Nurse, Their Workplaces, Number of Students Per Full-Time, and Education.

	School nurses *n *= 10 (%)
Gender	
Female	10 (100)
Male	0 (0)
Year working as a school nurse	
0–2 years	0 (0)
3–5 years	6 (50)
6–10 years	3 (30)
11–15 years	0 (0)
≥16 years	1 (10)
Workplaces	
Primary school with children aged 6–12	2 (20)
Secondary schools with adolescents aged 13–16	3 (30)
Upper secondary school with adolescents aged 16–19	5 (50)
Adapted school was included for 2 of the school nurses	
The number of students the school nurse is responsible for on a full-time basis (40 h/week)	
≤300 students	3 (20)
301–500 students	6 (60)
501–700 students	1 (10)
Subjects in their Master of Science in Nursing	
Pediatric nurse	4 (40)^a^
Primary Health Care	5 (50)^a^
School nursing^b^	1 (10)
Another specialist nursing program	1 (10)

^a^ One nurse has a master's in both Pediatric Nursing and Primary Health Care.

^b^ One university in Sweden has a specialist nursing program that is exclusively for school nursing.

### Procedure and Data Collection

From January to March 2024, the school operations managers and then school nurses were contacted and informed about the study orally and in writing to obtain their consent to participate. To facilitate participation for the school nurses, the time for the interview was adapted to their schedule. As the informants were in different parts of the country, all interviews were conducted via online video call and were between 17 and 52 min long. The first interview was conducted as a pilot test to evaluate the quality of the interview guide (Polit & Beck, 2021). One question was removed because it was not considered open-ended and overlapped with another. The interview guide, including 11 open-ended questions, was used throughout the semistructured interviews. Four examples of questions are: How do you discover whether a child lives in a culture of honor? What opportunities and difficulties do you experience when meeting a child who lives in a culture of honor? How do you feel about your knowledge of the culture of honor? What aspects of the culture of honor do you wish you knew more about? To encourage further elaboration, the open-ended follow-up questions, “How?” and “When?” and “Can you give an example?” were used. MQ or EE conducted the interviews and transcribed them verbatim.

### Data Analysis

Data were analyzed using qualitative content analysis with an inductive approach (Graneheim & Lundman, 2004). According to Graneheim and Lundman's (2004) method, a manifest content analysis was used to analyze the interviews. A manifest analysis involves remaining closely aligned with the data. In the first step of the analysis process, the data in the form of the original transcript were read repeatedly in their entirety. In the second step, the data were decontextualized into meaning units. Meaning units are parts of words, sentences, or paragraphs of the text that relate to the aim of the study. In the third step, the meaning units were condensed, which meant shortening the text but still retaining its content, then coding it. Coding means labeling the condensed meaning unit with a descriptive code close to the original text and on a low level of abstraction and interpretation (Lindgren et al., 2020). After that, the codes were carefully sorted into groups based on similarities and differences during recontextualization. Then, three categories and eight subcategories were created after an abstraction and interpretation of the text while maintaining the manifest content. Finally, an overarching theme emerged as a unifying “red thread” running through the subcategories and categories (Table 2).

**Table 2.** Examples of How the Analysis Was Conducted.

Meaning units	Condensed units	Code	Subcategories	Category	Theme
Exactly, yes, and you must ask the questions. Somehow, you can ask for permission, right? May I ask a question that I have been thinking about? Then you have a little respect and ask for permission	Must ask the questions. Ask for permission to ask. Respect and ask for permission	To dare to ask the question	Daring to meet the child	Building trust in children	Walking a tightrope when supporting children in honor cultures
It is difficult because of the law of secrecy. For example, I have been told I cannot talk to the school principal. I must talk to my coordinating school nurse in that case. So, I have backing to a certain extent, but I feel lonely because of the confidentiality I must maintain at school	It is difficult because of the law of secrecy. I feel lonely because of the confidentiality I must maintain at school	Confidentiality between staff can make it more difficult	Cooperation within the school	Collaborating around the children	

### Trustworthiness

To ensure trustworthiness, the study followed the criteria established by Lincoln and Guba (1985): credibility, dependability, confirmability, and transferability. Triangulation ensured credibility, where multiple researchers analyzed the data and compared their interpretations. Furthermore, to decrease the risk of missing essential content, the original transcript was read in parallel, thus ensuring its content was preserved. Continuous discussions within the research team strengthened the credibility of the findings. Dependability was achieved by maintaining a detailed audit trail that documented all methodological decisions throughout the study. Throughout the analysis process, the researchers practiced reflexivity, acknowledging their own preconceptions and striving to minimize bias (Lincoln & Guba, 1985). Confirmability was addressed by ensuring that findings were grounded in the data, with clear links between raw data, codes, and categories. Therefore, the analysis process moved back and forth between the original text and its parts. The use of direct quotes from participants further enhanced confirmability. Transferability was considered by providing a detailed description of the study context, participant characteristics, and data collection methods, allowing readers to assess the applicability of the findings to other settings (Lincoln & Guba, 1985).

### Ethical Considerations

This study adhered to established ethical research principles by ensuring compliance with requirements concerning respect for autonomy, nonmaleficence, beneficence, and justice (Good Research Practice, 2024; World Medical Association, 2013). Before participating, all the school nurses received both oral and written information detailing the study's purpose and procedures, and their rights as participants. Information and consent letters were prepared in accordance with the Swedish Ethical Review Authority's guidelines.

Participation was entirely voluntary, and informed consent was obtained from all participants. To safeguard confidentiality, all collected data were securely stored on password-protected computers, and any personally identifiable information was removed to ensure anonymity. In accordance with ethical guidelines, the data were used solely for the purposes explicitly outlined in the consent process. Additionally, the researchers remained attentive to ethical considerations throughout the study, and ensured that the participants felt comfortable and respected during the interviews.

## Results

Three categories described the school nurse's experience of meeting children living in a culture of honor: working preventively around children, building trust in children, and collaborating around the children (Table 3). Each category included two to four subcategories. As a “red thread,” an overarching theme emerged from the subcategories and categories, describing the school nurse's meeting with children living in a culture of honor as walking a tightrope.

**Table 3.** Overview of Subcategories, Categories, and Theme.

Subcategories	Category	Theme
Advancing school nurses’ preventive role Developing the child's knowledge	Working preventively around children	Walking a tightrope when supporting children in honor cultures
Discovering the child Creating a relationship with the child Using health dialogs as a tool Daring to meet the child	Building trust in children	
Cooperation within the school Cooperation outside of school	Collaborating around the children	

### Walking a Tightrope When Supporting Children Living in a Culture of Honor

On an overarching level, the school nurses’ experience of meeting children living in a culture of honor could be described as walking on a tightrope. It reflected the delicate balance required in their interactions. School nurses described it as challenging but crucial to work preventively, build trust, and cooperate internally and externally. In each action, conversation, and meeting, they thought carefully about what they said and did—they needed to be constantly on their toes and not do something wrong. It was like walking a tightrope.

### Working Preventively Around Children

In the first category and its subcategories, the school nurses described a need to advance their preventive role around children in a culture of honor. This included enhancing their knowledge and educating the children about the body, honor, and children's rights.

#### Advancing School Nurses’ Preventive Role

School nurses described how they had acquired a “clinical skill” that allowed them to be more confident today than when they started their careers. They found it rewarding to work preventively around children living in a culture of honor. Although they had good knowledge of the culture of honor and the treatment of these children, they knew they had more to learn. For example, they were more comfortable with their knowledge of how to identify and support girls exposed to honor oppression, compared to boys in similar situations. The school nurses wanted more knowledge about what signs they should look for in both girls and boys, and more practical knowledge about how to engage with, talk to, and help children living in a culture of honor. They also found it challenging to discuss the culture of honor with the children, as they did not want to risk being perceived as discriminatory. In this case, the school nurses felt they had not received enough training in honor-related violence and the culture of honor, either in the nursing or specialist nursing programs. Based on their experience, the school nurses highlighted the importance of learning through experience and skills enhancement.

*“I would like more hands-on training … Examples of questions you can ask in the health dialogs. Or a question in a questionnaire? It is a neutral way of asking … such as the question about genital mutilation that all children get.” (Informant 9)*

#### Developing the Child's Knowledge

The school nurses felt that children they suspected were exposed to honor norms lacked knowledge about the body's biology and their rights. Therefore, when the school nurses participated in teaching about sex, honor, and the child's rights, they tried to create discussions where the children had the opportunity to reflect on these topics. Still, expressing themselves correctly could be perceived as a balancing act. In the conversations, the children often talked about the norms and values they had brought from home. For example, it is against the law for a parent to hit their child in Sweden, but it might be okay in the child's home country. When the children's norms and values became visible in the teaching session, the school nurses could discuss these in an individual conversation later. Furthermore, the school nurses experienced frustration and powerlessness over being unable to reach and help these children as they would have liked. At the same time, they emphasized the importance of being present and persistent and believed that every tiny seed sown gave hope and might spark thought and increase the vulnerable child's knowledge.

*“We were in class and talked about norms, rules, and laws. These can look very different depending on where you come from. We asked the children, “Is it okay to hit your child?” A boy said, “Yes, you should do that because then you are a good father.” (Informant 1)*

### Building Trust in Children

The school nurses described the need to build trust with the children in the second category and its subcategories. They found it necessary to genuinely be in the moment and see the student, listen carefully, and dare to ask difficult questions to discover more about them. A good relationship had to be established to access the children's stories.

#### Discovering the Child

The school nurses described the difficulties they encountered when identifying children living within a culture of honor; it was often just an intuitive feeling that arose. Sometimes, the children repeatedly sought help from the school nurse for physical problems, such as stomach pain, headaches, or dizziness. They might also ask to be weighed or have their height measured or might come in to talk. The repeated visits gave the school nurses a gut feeling that something was wrong, leading them to start detective work to see if their suspicions were justified. When the school nurses brought up the subject of honor with the children, the typical response was that everything was fine. However, the impression was that the children were often aware of what they were and were not allowed to say. The school nurses noticed differences in their work with younger children, adolescents, and children with intellectual disabilities in adapted schools. They observed that younger children and those in adapted schools tended to speak more openly and lacked the filters that adolescents often have. The school nurses believed this difference might be because adolescents’ lifestyles are more limited and controlled, affecting their willingness to speak freely.

*“I can feel that it is almost easier when the children are younger. They are more transparent then, and when they get older, they fall silent. They then know what to say and not … and what may not be completely okay according to Swedish standards.” (Informant 1)*

#### Creating a Relationship With the Child

According to the school nurses, discovering and reaching children living in a culture of honor is complex because they have grown up in a culture of silence and are afraid of embarrassing their families. To reach the children, relationship-building work is required, where accessibility and trust are crucial. The school nurses said building relationships took a long time and was sometimes difficult. They described the importance of being available, having the door open, and always welcoming the child. Many said they scheduled follow-up conversations about somatic problems, such as sleep problems, when the child did not confide in them at the first visit because the child might need several conversations before daring to trust the school nurse. The school nurses said that getting close to the children and getting them to talk about their situation was difficult, as they knew the consequences for themselves and their families. Despite the difficulties of getting close to these children, it was essential not to give up. The school nurses described the importance of the children knowing that the school nurse was working to help them with other factors that affected their health.

*“They [the children] learn early on that they should not talk about anything that has to do with their family with people outside the family … because it is extremely taboo. I think that's why it usually takes time [before the child opens up]; you need to build up trust and build a good relationship with the child.” (Informant 6)*

#### Using Health Dialogs as a Tool

The school nurses highlighted the possibility of using the health dialogs as a tool to discover the children who lived in a culture of honor. According to the school nurses, health dialogs are the first step in creating a meaningful relationship with the child. During the health dialogs, the school nurse is alone with the child, which can allow the child to open up. Before the health dialogs, the child is asked to answer questions about their health, including violence and whether someone has done something to them against their will. At some schools, they even include questions about honor. The school nurses said that children they suspected lived in a culture of honor rarely explicitly discussed it. However, the school nurses always invited the child to talk about the culture of honor during health dialogs if they wished to do so.

*“It can be in the health dialogs in grade 8. Our survey has a direct question: Are you exposed to honor-related oppression? Virtually everyone answers ‘no’ to it. So, you need to … to read between the lines.” (Informant 4)*

#### Daring to Meet the Child

The school nurses emphasized the importance of nonjudgmentally interacting with children from cultures of honor, focusing on listening and responding to the child's needs. If a school nurse noticed significant changes in a child's behavior, appearance, or social relationships, they might feel something was wrong. In such cases, it was essential to show courage to ask difficult questions and offer support, especially with children who felt limited by a culture of silence. The school nurses emphasized to children that they were available to discuss sensitive or challenging topics and made it clear that their role was not to judge or blame them. They strove to build trust by emphasizing confidentiality and making the child feel comfortable about coming forward when ready.

*“It is a challenging area [to meet children living in a culture of honor]. Nevertheless, we can never give up, and we must dare and try to improve things, even if it is challenging to do so.” (Informant 2)*

### Collaborating Around the Children

The third category, and its subcategories, states that collaborating around the child is the most beneficial method of working with children who live in a culture of honor. School nurses often felt a deep concern for children subjected to a culture of honor, which motivated them to take proactive measures whenever possible and report their concerns further when necessary. They found that a collaboration that integrated efforts from multiple roles within the school and external authorities to provide holistic support to the child was most effective.

#### Cooperation Within the School

The school nurses described a collaboration to support the child involving many of the school's professionals, such as the student health team, mentors, and teachers. The cooperation and togetherness around the child were valuable because many professionals saw the child from their own point of view. This could be important in providing several pieces of the puzzle representing the child's mental, physical, and social health and the home situation. According to the school nurses, cooperation within the school was essential for detecting, helping, and supporting children exposed to a culture of honor. Despite this, school nurses could feel alone in their profession as they had the most robust confidentiality, which prevented them from expressing their concerns about the child to other professional groups at school. In addition to the confidentiality, the school nurses experienced inadequate routines. The loneliness of the profession and inadequate routines led to a feeling of hopelessness and frustration among the school nurses.

*“It is difficult because of the law of confidentiality. For example, I have been told I cannot talk to the school principal [about a child I am concerned about]. If I need to, my coordinating school nurse is the only one I can speak to. So, I have support to a certain extent externally, but I feel lonely because of the confidentiality I must maintain at school.” (Informant 9)*

#### Cooperation Outside of School

The school nurses discussed doing everything for the children exposed to the culture of honor. They believed collaboration with actors outside the school was crucial when trying to help such children. The collaboration described could involve working together preventively and reporting to external contacts responsible for further measures. The actors that the school nurses collaborated with included the social services, the women's shelter, the resource center, child and adolescent psychiatric service, and the police. Although the school nurses knew they had an obligation to report to social services when they suspected a child was living in a culture of honor, they faced significant challenges. They understood the importance of reporting their concerns but feared their actions could worsen the child's situation. The school nurses also mentioned occasions when they felt threatened by the child's family. The fear accompanying the threat caused uncertainty about whether to report the family to the social services, even if they knew they had to. In addition, the school nurses said that for social services to take their concerns seriously, they often needed to provide more documentation than in other child protection cases. This increased expectation could complicate the reporting process and make school nurses uncertain whether their reports would lead to action. Another obstacle that the school nurses mentioned regarding collaboration with the social services was the confidentiality that the social services maintained toward the school nurses. The school nurses felt it was difficult to handle the children's everyday situations at school after they had written a mandatory report of concern and had not received feedback from the social services. Sometimes, school nurses helped children get in touch with social services, which could help them get a protected identity or move away from their families. However, some children returned to their families and were in the same situation a few months later. This created uncertainty among the school nurses about whether their actions had improved the situation. The school nurses also contacted the social services if the girls had sought out the school nurse before planned trips because they were worried about being married off, or if the girls had disappeared during the summer holidays. After being contacted, the social services sometimes told the school nurses they could not do anything. They could not prevent the child and the family from going abroad. This created a feeling of powerlessness in the school nurses.

*“We understand that she has a one-way ticket. Nevertheless, it is not forbidden to travel abroad, and we cannot do anything about it, they [the social services] answered. They could not say much more. They have strong confidentiality.” (Informant 4)*

## Discussion

The study aimed to describe the school nurses’ experience of meeting children living in a culture of honor. The results show that the school nurses find their work meaningful but challenging and sometimes frustrating. They are uncertain whether their efforts improve or worsen the child's situation. The importance of working preventively, building trust, and collaborating with other professionals is particularly emphasized.

The study reveals that school nurses’ knowledge of honor-related violence varies. They gain knowledge through lectures and experience but express a need for more concrete tools and clear routines. This suggests that while school nurses may be well-versed in recognizing signs of honor-related violence, there are still gaps in their knowledge and the resources available to help them manage these cases effectively. Previous research has shown that school nurses with extensive experience and training feel more comfortable identifying and acting on their intuition, further emphasizing the importance of continuing professional development (Harding et al., 2019). Bhatia et al. (2024) similarly noted that gaps in resources and knowledge hinder school nurses’ ability to effectively address honor-related violence, underscoring the need for structured support. Focusing on targeted educational interventions, such as scenario-based simulations and interdisciplinary workshops, could enhance school nurses’ ability to respond effectively to honor-related violence (Gregory et al., 2020). Additionally, Engh et al. (2024) highlighted that systematic screening for child maltreatment by school nurses facilitates disclosure, underlining the need for structured interventions to identify children at risk.

Reflecting previous research, school nurses often notice that girls are more vulnerable in the culture of honor (Strid et al., 2021). However, the importance of considering boys’ experiences has also been emphasized. Boys, too, may experience violence and coercion in a culture of honor, including the pressure to monitor their sisters. To better support boys in these situations, school nurses must develop approaches that identify and address the unique challenges boys face in a culture of honor (Wilhsson et al., 2023).

A key approach for school nurses identified in previous research is informing children about their rights, as this is essential in helping them seek support (Gregory et al., 2020; Silivri et al., 2023; WHO, 2021). The results of this study also show that collaboration with other professionals within and outside the school is crucial for improving the child's situation. School nurses emphasize the importance of working closely with the student health team and teachers, as well as external agencies like the police and social services. Effective communication with social services increases their confidence in reporting concerns. However, many school nurses hesitate to make mandatory reports, fearing it might worsen the child's situation. This fear of worsening the situation is in line with previous research (Högdin et al., 2023; Sundler et al., 2021), which also showed a lack of trust in the social services’ knowledge or ability to support children living in a culture of honor (Högdin et al., 2023). This may induce uncertainty or an internal moral conflict when reporting to social services. To strengthen school nurses’ confidence in reporting concerns, it is important to provide clear guidelines and increase organizational support for their role in child protection (Sundler et al., 2021; Widmark et al., 2011).

Building trusting relationships with the children is another aspect emphasized by the school nurses, which aligns with previous studies (Alizadeh et al., 2011; Harding et al., 2019). Children may initially deny being subjected to violence but later report it to the school nurse, making long-term follow-up crucial (Gregory et al., 2020). Engh et al. (2024) further emphasized that structured follow-ups after initial screenings can be instrumental in ensuring that children feel safe enough to disclose their experiences. The school nurses describe their detective work to identify at-risk children and the frustration when some children view honor norms as normal. To address children who do not perceive their situation as problematic, school nurses should adopt strategies that focus on gradually building trust and should use sensitive questioning.

### Implications for School Nursing

Children living in a culture of honor are a particularly vulnerable group. The school nurse's role in identifying and helping these children is crucial. The results suggest that school nurses need additional training, better organizational support, and more time to build relationships to manage these cases. It would also be valuable to explore whether certain challenges faced by school nurses have worsened or changed over time, distinguishing this study's findings from previous research. Further research is needed to investigate children's own experiences of school nurses’ interventions and their expectations of school health services. To enhance the effectiveness of school nurses, organizations should develop concrete strategies that provide more explicit guidelines and should allocate more resources to support school nurses in managing these complex cases.

### Strengths and Limitations

The trustworthiness of this study was ensured by including a diverse group of school nurses who all had experience dealing with children living in a culture of honor but worked within different socio-economic areas, geographical areas, and school years. The fact that the school nurses had solid experience strengthens the results, and the varied selection increases the study's credibility (Lindgren et al., 2020). Furthermore, increased credibility was ensured by using a critical and questioning approach in the data analysis, moving back and forth between the whole text and the meaning units, and discussing the results in seminars. According to Lincoln and Guba (1985), such methods align with the principles of maintaining credibility through rigorous analysis and reflexivity.

The fact that the study only included 10 school nurses may be seen as a weakness, although it is an acceptable number of participants in qualitative research. An additional limitation may be that the school nurses who participated in the study were more motivated and interested in the phenomenon than those who declined participation. This could impair the credibility and transferability of the study, as the selected informants may not represent the school nurses as a group. However, a structured and clearly described analysis process strengthens the transferability of the study (Polit & Beck, 2021; Lincoln & Guba, 1985). The authors have repeatedly processed and discussed the analysis, subcategories, and main categories in seminars to ensure dependability, and have reflected on the school nurses’ voices using quotes to ensure confirmability (Graneheim & Lundman, 2004). Lincoln and Guba (1985) further emphasize that these steps contribute to the overall trustworthiness of the study, and ensure that the findings are grounded in the data. However, the reader must decide on the transferability, and whether the results can be usefully applied to other settings, contexts, or situations (Lincoln & Guba, 1985).

## Conclusion

This study highlights school nurses’ challenges when supporting children living in a culture of honor. While they find their work meaningful, they often experience uncertainty, fearing their actions may worsen the child's situation. School nurses with more experience and who collaborate more closely with other professionals reported feeling more confident, but knowledge gaps remain. The findings emphasize the need for clearer guidelines, structured training, and interdisciplinary collaboration to support at-risk children effectively. The school nurses stressed the importance of preventive work, trust-building, and informing children about their rights. However, hesitation about reporting concerns to social services suggests the need for stronger organizational support. While girls were more frequently identified as victims, the study also highlights boys’ unique challenges. Future efforts should include gender-sensitive approaches. Strengthening school nurses’ competence, resources, and interprofessional collaboration is crucial to ensuring vulnerable children receive the protection and support they need.
